# Awareness Levels about Breast Cancer Risk Factors, Early Warning Signs, and Screening and Therapeutic Approaches among Iranian Adult Women: A large Population Based Study Using Latent Class Analysis

**DOI:** 10.1155/2014/306352

**Published:** 2014-09-11

**Authors:** Mahdi Tazhibi, Awat Feizi

**Affiliations:** Department of Biostatistics and Epidemiology, School of Health, Isfahan University of Medical Sciences, Isfahan 81746-73461, Iran

## Abstract

*Background and Objective*. Breast cancer (BC) continues to be a major cause of morbidity and mortality among women throughout the world and in Iran. Lack of awareness and early detection program in developing country is a main reason for escalating the mortality. The present research was conducted to assess the Iranian women's level of knowledge about breast cancer risk factors, early warning signs, and therapeutic and screening approaches, and their correlated determinants. *Methods*. In a cross-sectional study, 2250 women before participating at a community based screening and public educational program in an institute of cancer research in Isfahan, Iran, in 2012 were investigated using a self-administered questionnaire about risk factors, early warning signs, and therapeutic and screening approaches of BC. Latent class regression as a comprehensive statistical method was used for evaluating the level of knowledge and its correlated determinants. *Results*. Only 33.2%, 31.9%, 26.7%, and 35.8% of study participants had high awareness levels about screening approaches, risk factors, early warning signs and therapeutic modalities of breast cancer, respectively, and majority had poor to moderate knowledge levels. Most effective predictors of high level of awareness were higher educational qualifications, attending in screening and public educational programs, personal problem, and family history of BC, respectively. *Conclusion*. Results of current study indicated that the levels of awareness among study population about key elements of BC are low. These findings reenforce the continuing need for more BC education through conducting public and professional programs that are intended to raise awareness among younger, single women and those with low educational attainments and without family history.

## 1. Introduction

Breast cancer (BC) as a multifactorial disease is one of the most common cancers and the second leading cause of deaths among women worldwide [1]. Global statistics show that the annual morbidity and mortality of BC are increasing, in which over 1.15 million women worldwide (representing 10 percent of all diagnosed cancers and 23 percent of cancers diagnosed in women) are diagnosed with breast cancer each year and more than 502,000 of them die from this disease. The disease accounts for more than 1.6% of all female mortality worldwide [2–4].

Breast cancer is a major public health problem in developed nations and is becoming an increasingly predominant problem in low and middle income countries, where incidence rates have been increased by up to 5% per year [4, 5]. According to the World Health Organization, the incidence rates in the developing countries will rise because of increasing life expectancy, growing urbanization, and greater adoption of Western lifestyles [6].

In Eastern Mediterranean countries, breast cancer is the most common cancer and in Iran, breast cancer ranks first among cancers diagnosed in women, in which 76% of common malignancies among Iranian women are breast cancer comprising 1200 deaths per year with a crude incidence rate and age specific rate of 17.4 and 23.1 per 100,000 of female population, respectively [7, 8]. In Iran the incidence of the breast cancer is rising, patients present with advanced stage of disease and they are relatively younger (about 10 years; majority of patients population in Iran, that is, 86.1%, are <50 yrs), in which the age-specific incidence rate per 100,000 population varies between 33 and 74 among Iranian female aged 35–50 years old versus 10 to 20 for western women counterparts [1, 9–12].

The etiology of the majority of breast cancers is not known carefully, in which only about 25% to 40% of them may be attributed to well known risk factors [13]. The risk factors for breast cancer vary with respect to geographic characteristics and life-style-related habits of a community. However, numerous common risk factors for the disease have been established. These risk factors include female gender, increasing age, family history of BC, early menarche, late menopause, older age at first live childbirth, genetic mutation, diet, obesity, smoking, and alcohol consumption [5, 14]. Nutritional and epidemiological surveys have shown that dietary and lifestyle factors such as obesity, smoking, alcohol consumption, and sedentary lifestyle play significant role as risk factors for breast cancer while breast feeding practice is protective against breast cancer [15, 16].

Many of the breast cancer cases, in developing country, including Iran, are diagnosed in advanced stages, attributable to low level of awareness about early warning signs and screening methods among general women population and poor prognosis of the patients.

Various epidemiologic studies have shown that the increased women's knowledge about early diagnosis and screening of breast cancer can change people's screening seeking behavior [3, 17].

Early diagnosis of cancer can effectively improve the chance of early detection of breast cancer in early stages and successful treatment resulting in improvement survival rate and quality of life. In this regard, early detection of disease through clinical breast exams such as mammography and breast self-examination as simple and inexpensive approaches provides the best approaches for reducing the risk of dying from breast cancer. Accordingly, correct knowledge about early warning signs and screening methods of disease plays an effective role towards developing and employing early detection programs in a community [17].

Although biological factors consistently can be considered as key elements of final outcome, in the absence of breast screening programs, stage at presentation remains the most important prognostic indicator in regard to detecting breast cancer at a preclinical stage. This is more important when there is no established national screening registry and program for breast cancer [13].

In a community such as Iran where late presentation is predominant and majority of breast cancer patients are diagnosed at advanced stages of disease there is an urgent need for improving the levels of awareness about breast cancer and its early detection measures. The awareness about breast cancer among Iranian women is not well documented particularly among general public. Some limited studies in Iran have shown that the awareness level about risk factors and early detection measures of breast cancer such as clinical breast examination (CBE) and breast self-examination (BSE) is low [1, 11]. These studies have some major shortcomings including lack of a population based survey structure, and none of them analytically evaluated awareness levels. On the other hand, methods of primary prevention are rarely investigated, particularly via health promotion activities. Accordingly, baseline reports about level of knowledge would be vital to promote the prevention and early detection of breast cancer's activities, hence the need for implementing studies for assessing level of knowledge of breast cancer in the population [3, 17]. As we are aware, there is no study that, in Iran, evaluated comprehensively the awareness levels about risk factors, early warning signs, and therapeutic and screening modalities as well as the predictive factors for special characteristics associated with enrolment in breast cancer prevention.

The aims of current large population based survey among Iranian adult women were to evaluate the levels of knowledge about risk factors, early warning signs, screening approaches, and therapeutic methods of breast cancer and to determine which women are most at risk of less knowledgeable. There has previously been disagreement in the literature regarding the best ways to measure cancer's awareness, making it difficult to determine levels of awareness reliably. In current study, we applied a comprehensive and relevant analytical statistical method, that is, latent class analysis (LCA) and its extended form, that is, latent class regression (LCR) [18, 19] that provides a reliable descriptive perspective on public awareness about various aspects of breast cancer and their potential determinants.

## 2. Methods

### 2.1. Study Design and Participants

This large population-based cross-sectional study carried out among 2250 women interested in participating at a community based screening and public educational program about breast cancer in the cancer research institute in Isfahan, Iran, since January to March, 2012. The cancer research institute in Isfahan, Iran, using different invitation methods (including local television and radio's programs, billboard, etc.) invited people to participate in a planned comprehensive cancer control program. As a part of this population based program, the women's awareness levels about different aspects of breast cancer including risk factors, early warning signs, and therapeutic and screening approaches were evaluated. During the study period, a well face and content validated questionnaire (see Section 2.2) was distributed through a trained nurse among 2550 people who attended the institute before contributing at screening and educational programs. Finally, the information of 2250 people with no missing values was considered in data analysis. The study was approved by the ethic committee of Isfahan University of Medical Sciences. The participants were informed about the increased incidence rate of breast cancer, particularly among the young Iranian's female population. Written informed consent was obtained from all participants after complete explanation about the study objectives.

### 2.2. Study Instrument and Variables Assessment

A self-administered well face and content validated structured questionnaire was used for data collection. The questionnaire was organized in five distinct sections; each one documented with appropriate heading indicating its content, as follows: first section contained sociodemographic variables such as age, educational status (three categories including “illiterate, less educated, or less than 12 years formal education,” “12 years formal education,” and “university graduation”), personal breast problems history (Yes/No), first relatives' family history and marital status (Married/Single) attending in screening and public educational programs (Yes/No). The majority of the questions in other four sections of our study questionnaire were considered based on the American cancer society [20].

Second section contained questions about 10 well-established breast cancer's risk factors including: infertility, age at menarche, age at menopause, breastfeeding, family history of breast cancer, obesity, smoking, alcohol consumption, diet, and trauma; third section included 7 early warning signs including: lump in breast, nipple retraction, breast or nipple pain, discharge other than breast milk in nipple, breast asymmetry, lump or swelling under the armpit, redness, and scaliness or thickening of the nipple or breast skin; fourth section addressed questions about breast cancer screening methods including: biopsy, sonography or CT scan, mammography, monthly self-examination, and biannually breast examination by a physician; and fifth section requested information on evaluating the level of awareness about breast cancer therapeutic methods including: “surgery or removal of whole or part of breast” and “chemical or radiotherapy” and third item was “it depends on disease's stage.” All questions in above four study's instrument sections were recorded as “yes” and “no” options.

### 2.3. Statistical Analysis

Summary statistics were reported for study participants as mean ± SD for quantitative variables and percent for qualitative variables.

To analyze the level of “knowledge” about each studied domain of breast cancer, it was considered as a latent construct and was evaluated based on having knowledge or lack of knowledge about each item included in each domain using latent class analysis (LCA) and the determinants of knowledge were evaluated using extended form of LCA, that is, “latent class regression” analysis (LCR). Latent class analysis (LCA) examines the pattern of relations among a set of observed categorical variables and identifies and classifies similar individuals into latent classes [18]. This leads subjects within each latent class which are highly similar to each other and uniquely different from the other classes across the set of evaluated variables. Accordingly, comparisons can be made across latent classes with regard to correlates and other adjustment variables. It is useful to include covariates in the LCA (i.e., latent class regression or LCR). LCR still can find homogeneous groups of individuals, but now covariates are included to describe both the formation of the latent classes and how they may be differently measured by the observed indicators. The prediction of latent class membership is obtained by multinomial regression of latent class variable on covariates [19].

All descriptive and analytical analyses in the present study were performed with “*R*” (version 3.0.2): an open source comprehensive statistical package.

## 3. Results 

The mean (±SD) and range of age for the study participants were 36.8 (±9.1) and 15–67 yrs. 51.9% of study participants had university educational attainment and 81.9% of them were married. 92.5% and 81.9% of study participants had no personal breast problems history and first relatives' family history, respectively.

Table 1 shows the prevalence of the correct answers to the questions about specific breast cancer screening approaches in constructed classes. The nature of each class can easily be interpreted in terms of awareness levels about the screening approaches. Accordingly, class 1 contains 33.2% of the study population and included individuals with high knowledge level and class 3, including 34.9% of participants, consisted of people with low awareness level. The second class included individuals with mixed situation in terms of awareness levels about cancer screening approaches.  Table 2 provides information about the relative impacts of each studied determinant of knowledge levels about breast cancer screening approaches in terms of latent class regression coefficients. In this regression method the constructed classes play the role of categories of dependent variable and similar as ordinary logistic regression one of the categories (classes) is chosen as the reference category and the covariates coefficients can be interpreted as odds or log odds ratios of being an individual in a specific category instead of reference class. Here, the third class was considered as a reference category; therefore the investigation of covariate coefficients show that university educational attainment, positive family and personal history, and contributing in educational screening and preventing program have positive impacts for being an individual in higher levels of awareness (OR > 1, *P* < 0.05). As can be seen from Table 2, majority of studied covariates did not have significant impact on distinguishing of class 2 from class 3.

Applying latent class analysis for evaluating of knowledge levels about 10 well known breast cancer risk factors lead to three classes (see Table 3). According to prevalence of correct answers to considered risk factors in constructed classes, the first class included people with high knowledge, second class and third class consisted of individual with moderate and low levels of awareness, respectively. The size of classes indicates that the most of respondents were in moderate and low levels of knowledge.  Table 4 shows the relation of studied determinants with level of awareness. The third class was considered as reference category; according to covariates' coefficients in first class level of education (university category; OR = 3.9, *P* < 0.01), personal history (OR = 5.06, *P* < 0.05), and attending in educational and screening programs (OR = 3.08, *P* < 0.05) were significantly related with high levels of awareness. Married and older women were marginally more aware (*P* < 0.1).

Table 5 presents the prevalence of knowledge level about warning signs of breast cancer, as measured by “yes” responses to seven signs. Two distinct classes were identified using latent class regression. As Table 5 shows, class 1 included the individuals with higher knowledge level while class 2 consisted of the individuals with lower awareness level. According to class's size, only small proportion of the respondents (26.7%) had high level of knowledge and most of them were included in class 2 (71.3%).  Table 6 contains the estimates of latent class regression coefficients. In LCR model class 2 was considered as reference category. As can be seen, the effective factors on level of awareness about warning signs, in order of importance, were contributing in screening and educational program (OR = 2.34, *P* < 0.01), educational attainment level (OR = 1.96, *P* < 0.01), personal (OR = 2.32, *P* < 0.1) and family (OR = 1.69, *P* < 0.05) history, and marital status (OR = 1.64, *P* < 0.01).

In current research, awareness about three major therapeutic approaches (see Table 7) for breast cancer was investigated. Two distinct classes from studied participants were identified using Latent class regression analysis. Examining the observed conditional correct response probability (Yes) for the 2-class model in Table 7 shows a class of people who were basically not aware about any of the therapeutic methods (35.8%) and a class included individuals with high probability of correct response to every therapeutic modality (64.2%). The results of latent class regression are presented in Table 8. Second class was considered as a reference category. As Table 8 shows that the statistically significant effective covariates on high awareness level were educational attainment (OR = 3.6, *P* < 0.0001), attending in screening and educational programs (OR = 2.58, *P* < 0.01). Personal and family history was marginally positively correlated with high levels of awareness.

## 4. Discussion and Conclusions

Breast cancer as a multi-etiology disease has created a significant health problem worldwide. Breast cancer is the third common of all female cancer in Iran [12, 20]. Its incidence in Iran has risen significantly over the last two decades [12, 20] and is expected to continue to rise sharply through the years. The basic level of cancer knowledge of the population about diagnostic tools, screening, new approaches to prevention, early diagnosis, and treatment modalities is important for controlling cancer particularly breast cancer. Early detection of breast cancer plays the leading role in reducing mortality rates and improving the patients' prognosis [17].

This study was conducted to evaluate levels of awareness and their correlated determinants about risk factors, early warning signs, screening methods for early detection, and treatment of breast cancer in a large representative sample of Iranian women.

Results of current study showed that the level of awareness about breast cancer screening methods among study population was low, in which only 33.2% had high knowledge and remaining had mixed or weak awareness level. Also our study showed inadequate knowledge about well-known risk factors for breast cancer among study population, in which only about 32% of participants had high knowledge and majority of them (about 43%) had moderate and the remaining had low awareness levels. Age at menarche and infertility were the least frequently identified correctly as the risk factors for breast cancer in our study. The current study's results revealed that the participants had inadequate knowledge about breast cancer early warning signs in which the vast majority of them (71.3%) were in class with low levels of awareness. Although we evaluated awareness about well-known and widely used therapeutic methods, the results clearly indicated that knowledge levels among study population were low, in which only about 36% of them were in class with high levels of awareness. Although previous studies in this field did not provide aggregate and reliable features of awareness as we did in current study using latent class analysis, however they have shown poor knowledge, but slightly higher, about screening methods [1, 11, 21, 22] consistently about risk factors [23–28] a in developing as well as developed countries. Our findings regarding to awareness about early warning signs are similar to that of previous studies [27–29] but in contrary to the findings of some others one [27, 30]. However, review of the published literature showed that few numbers of studies have evaluated awareness about breast cancer's therapeutic options. In a study conducted among undergraduate Nigerian females 65.4% had good scores of knowledge [30], while in another one 26.7% of the study participants believed that mastectomy is the only treatment option and 54.9% did not any information about various therapeutic methods for breast cancer [31].

In terms of individual specific items in the area of breast cancer risk factors, some earlier studies provided similar results to our findings [23–26] and some others were reported lower [21, 28–32] or higher levels [27, 30] compared to our results. It is worth to notice that those people with overly high levels of knowledge about screening methods had poor correct identification of sonography and biopsy. The low proportion of the respondents acknowledged sonography and biopsy as an early detection measures can be attributed to not availability to this population particularly biopsy and more common applicability of sonography for other diseases than breast examination.

Generally, comparing the findings obtained in developed and developing nations did not clearly suggest obvious differences; although, in average, levels of awareness were higher in former ones. Majority of the observed differences about knowledge of all aspects of breast cancer can be attributed to specific sociocultural characteristics of studied populations.

The most effective predictors of knowledge level about breast cancer risk factors, screening methods, early warning sign, and therapeutic modalities were educational qualification, personal history and contributing in educational and screening public programs, respectively. The educational qualification was the common significant predictor for high awareness levels about all four studied domains. The observed important role of educational status on awareness level were in agreement with previous studies in screening approaches [23, 33–36], risk factors [37–40], early warning signs [21, 26, 27, 41, 42]. It is important to be mentioned we observed a more highly significant association between higher educational qualification and awareness level about therapeutic modalities compared to other three aspects. Although it is naturally expected that people having higher levels of education have more capability for obtaining more and effective information from various sources but knowledge about therapeutic options compared with risk factors, screening methods or warning signs may more influenced by educational qualification as we saw in our study that those people with university educational and high school educated people compared with illiterate or less educated ones had higher chance for being in class with higher levels of awareness.

Our results in line with other previous studies showed a positive association (OR > 1) between high awareness levels about screening methods [23, 33–36], risk factors [23, 43] and early warning sign [21, 42] with family and personal history and participating in screening and educational public programs. The effective role of formal training programs and correct structured theoretical educations on breast cancer awareness and BSE training on improving of awareness levels about various aspects of a disease cannot be denied [11]. Although, in few studies the significant association was not observed between family or personal history and awareness level about BC risk factors and screening methods [44], however the consistent findings on the association of personal history or family history of with awareness about breast cancer reconfirm the importance of their predictive roles. It seems that a personal or family history of cancer could be expected to be associated with different causal beliefs; it is expected those educated and with a family or personal history of BS were particularly likely to acknowledge the potential various “controllable” risk factors [45].

The present study found marginally significant association between age and awareness about BC's screening methods and risk factors, also results indicated that unmarried women had marginally lower knowledge about BS's risk factors while significantly lower knowledge about BC's screening methods. Similar results with our finding in these regards were found in some previous studies [23, 33–36, 46]. An explanation regarding these findings might be that married and older people are generally more concerned about their health due to being more at risk for chronic disease and higher responsibilities toward the family, respectively. Therefore, they have more capability for obtaining more and effective information from various sources about health determinants and in our study about breast cancer.

## 5. Study Strengths and Limitations

The present study can be considered as the first comprehensive research established in not only Iran but also over the world in order to investigating the public knowledge level about the various aspect of breast cancer. The second and most important strength aspect of current study was applying a sophisticated statistical method that provides a real and reliable descriptive perspective about women public awareness about breast cancer and its determinants. Previous studies, in our view, suffer from the partiality of the models and frameworks regarding to the evaluating of actual knowledge and its determinants among general public.

An apparent limitation of the current study was that the awareness levels of those people who are interested in attending at a public screening and educational programs about breast cancer in an cancer research institute was investigated which could reflect selection bias and resulting an over estimate of awareness levels about breast cancer's key issues. However, it should be noted that the awareness levels assessment was conducted before participating of responders in public screening and educational programs.

## 6. Concluding Remarks

In conclusion, the results of present study revealed low levels of awareness regarding breast cancer risk factors, early warning signs, and its screening and therapeutic approaches among a relatively large sample of Iranian adult women. The results also indicated that the women with higher educational attainments, attending screening and public educational programs, personal problem, and family history of breast cancer, had higher levels of awareness. These findings emphasis on raising awareness about breast cancer among Iranian women as an effective way to overcome a challenging important problem in Iran's public health scope, that is, increasing trend and burden of breast cancer disease. Awareness would lead to early detection and reduce the stage at diagnosis, potentially improving the odds of survival and cure with simpler and more cost effective treatment. Also, our findings will help in directing breast cancer public educational programs. Developing of public screening and educational program through health care system more emphasis on low educated, single, younger women and those without family history are needed among Iranian women.

## Figures and Tables

**Table 1:** Class-specific level of correct awareness (%) about the breast cancer screening approaches.

Screening approaches	Class 1 (high awareness)		Class 2 (mixed awareness)		Class 3 (poor awareness)	
	Yes	No	Yes	No	Yes	No
Biopsy	54.15	45.85	0.12	99.88	0.97	99.03
Sonography or CT scan	59.94	40.06	17.37	82.63	0.10	99.9
Mammography	87.63	12.37	47.14	52.86	4.76	95.24
Breast self-examination monthly	83.86	16.14	83.58	16.42	4.08	95.92
Breast examination biannually by a physician	90.32	09.68	76.70	23.30	5.60	94.40

Class size	33.2%		31.9%		34.9%	

**Table 2:** Multivariable odds ratio (OR) and 95% confidence interval (95% CI OR) of OR for the association of potential determinants of awareness level about screening approaches of breast cancer.

Class (reference: class 3)	Independent variables	Coefficients	*z*-value (*P* value)	Odds ratio (OR)	95% CI OR
Class 2	Age	0.02 (0.013)	1.59 (0.11)	1.02	(0.99, 1.03)
	Marital status (married)	0.24 (0.3)	0.80 (0.42)	1.27	(0.58, 2.67)
	Level of education^1^				
	12 years formal education	0.15 (0.38)	0.40 (0.68)	1.16	(0.54, 2.49)
	University graduate	0.46 (0.29)	1.61 (0.11)	1.58	(0.77, 3.13)
	Family history (Yes)	0.23 (0.35)	0.65 (0.52)	1.25	(0.57, 2.77)
	Personal history (Yes)	0.45 (1.36)	0.33 (0.74)	1.56	(0.70, 2.96)
	Attending in screening and education program	0.79 (0.43)	1.83 (0.07)	2.22	(0.91, 5.37)

Class 1	Age	0.03 (0.016)	1.93 (0.05)	1.03	(0.99, 1.04)
	Marital status (married)	0.13 (0.33)	0.40 (0.69)	1.15	(0.54, 2.43)
	Level of education^1^				
	12 years formal education	0.01 (0.24)	0.042 (0.97)	1.01	(0.98, 1.02)
	University graduate	0.62 (0.36)	1.73 (0.08)	1.86	(0.99, 2.83)
	Family history (Yes)	0.62 (0.26)	2.41 (0.012)	1.86	(1.21, 2.54)
	Personal history (Yes)	1.64 (0.55)	2.98 (0.003)	5.14	(1.20, 22.43)
	Attending in screening and education program (Yes)	0.88 (0.35)	2.49 (0.01)	2.44	(1.42, 4.39)

^1^Reference category is less than 12 years formal education, less educated and illiterate people.

**Table 3:** Class-specific level of correct awareness (%) about breast cancer's risk factors and the size of classes.

Breast cancer risk factors	Class 1 (high awareness)		Class 2 (moderate awareness)		Class 3 (poor awareness)	
	Yes	No	Yes	No	Yes	No
Age at Menarche	25.11	74.89	5.17	94.83	1.29	98.71
Diet	91.61	8.39	78.62	21.38	25.99	74.01
Age at menopause	93.17	6.83	78.91	21.09	30.21	69.79
Family history	66.42	33.58	32.81	67.19	5.53	94.47
Infertility	60.73	39.27	19.76	80.24	0.15	99.85
Obesity	92.55	7.45	42.57	57.43	0.10	99.00
Smoking	98.25	1.75	57.01	42.99	0.51	99.49
Alcohol consumption	97.31	2.69	47.77	52.23	0.46	99.54
Breast feeding	83.25	16.75	36.41	63.59	4.78	95.22
Trauma	73.58	26.42	31.32	68.68	7.60	92.40

Class size	31.9%		42.9%		25.2%	

**Table 4:** Multivariable odds ratio (OR) and 95% confidence interval (95% CI OR) of OR for the association of potential determinants of awareness level about breast cancer's risk factors.

Class (reference: class 3)	Independent variables	Coefficients (SE)	*z*-value (*P* value)	Odds ratio (OR)	95% CI OR
Class 2	Age	0.003 (0.01)	0.29 (0.78)	1.003	(0.98, 1.03)
	Marital status (married)	0.52 (0.34)	1.54 (0.12)	1.69	(0.87, 3.29)
	Level of education^1^				
	12 years formal education	0.23 (0.40)	0.56 (0.58)	1.25	(0.57, 2.77)
	University graduate	0.15 (0.39)	0.39 (0.69)	1.17	(0.54, 2.52)
	Family history (Yes)	0.21 (0.37)	0.56 (0.58)	1.23	(0.60, 2.53)
	Personal history (Yes)	0.87 (0.87)	1.04 (0.31)	2.39	(0.44, 13.07)
	Attending in screening and education program (Yes)	0.67 (0.59)	1.15 (0.25)	1.96	(0.62, 6.17)

Class 1	Age	0.03 (0.01)	1.76 (0.08)	1.03	(1.00, 1.05)
	Marital status (Married)	0.54 (0.29)	1.83 (0.07)	1.72	(0.96, 3.07)
	Level of education^1^				
	12 years formal education	0.10 (0.37)	0.28 (0.78)	1.10	(0.54, 2.28)
	University graduate	1.36 (0.40)	3.37 (0.003)	3.90	(1.77, 8.61)
	Family history (Yes)	0.007 (0.33)	0.021 (0.98)	1.007	(0.53, 1.92)
	Personal history (Yes)	1.62 (0.77)	2.20 (0.03)	5.06	(1.20, 21.43)
	Attending in screening and education program (Yes)	1.13 (0.51)	2.21 (0.03)	3.08	(1.14, 8.37)

^1^Reference category is less than 12 years formal education, less educated, and illiterate people.

**Table 5:** Class-specific level of correct awareness (%) about breast cancer's early warning signs and the size of classes.

Warning signs	Class 1 (High awareness)		Class 2 (poor awareness)	
	Yes	No	Yes	No
Breast asymmetry	75.56	24.44	13.76	86.24
Breast or nipple pain	73.67	26.33	24.05	75.95
Nipple retraction	75.65	24.35	7.95	92.05
Redness, scaliness, or thickening of the nipple or breast skin	73.05	26.95	1.28	98.72
Lump or swelling under the armpit	90.8	9.2	24.66	75.34
Bleeding or discharge other than breast milk in nipple	86.56	13.44	12.6	87.4
Lump in breast	95.73	4.27	37.01	62.99

Class size	26.7%		73.3%	

**Table 6:** Multivariable odds ratio (OR) and 95% confidence interval (95% CI OR) of OR for the association of potential determinants of awareness level about early warning signs of breast cancer.

Independent variables^1^	Coefficients (SE)	*z*-value (*P* value)	Odds ratio (OR)	95% CI OR
Age	0.008 (0.009)	0.87 (0.39)	1.008	(0.99, 1.03)
Marital status (married)	0.49 (0.20)	2.48 (0.01)	1.65	(1.11, 2.44)
Level of education^2^				
12 years formal education	0.24 (0.24)	0.97 (0.33)	1.27	(0.78, 2.04)
University graduate	0.67 (0.22)	3.02 (0.003)	1.96	(1.27, 3.02)
Family history (Yes)	0.53 (0.23)	2.24 (0.03)	1.69	(1.07, 2.67)
Personal history (Yes)	0.84 (0.47)	1.80 (0.07)	2.32	(0.93, 5.77)
Attending in screening and educational program (Yes)	0.88 (0.29)	2.96 (0.003)	2.40	(1.34, 4.29)

^1^Class 2 was considered as reference category in LCR model.

^2^Reference: Less than 12 years formal education, less educated or illiterate.

**Table 7:** Class-specific level of correct awareness (%) about the breast cancer's therapeutic modalities and the size of classes.

Therapeutic Approach	Class 1 (high awareness)		Class 2 (poor awareness)	
	Yes	No	Yes	No
Chemotherapy and radiotherapy	74.74	25.26	14.21	85.79
Mastectomy	71.76	28.24	1.04	98.96
Depends on disease's stage	98.24	1.76	19.88	80.12

Class size	35.8%		64.2%	

**Table 8:** Multivariable odds ratio (OR) and 95% confidence interval (95% CI OR) of OR for the association of potential determinants of awareness level about therapeutic approaches.

Independent variables^1^	Coefficients (SE)	*z*-value (*P* value)	Odds ratio (OR)	95% CI OR
Age	−0.005 (0.01)	−0.49 (0.63)	0.99	(0.98, 1.01)
Marital status (married)	0.25 (0.21)	1.21 (0.23)	1.28	(0.85, 1.93)
Level of education^2^				
12 years formal education	0.63 (0.25)	2.52 (0.01)	1.87	(1.15, 3.07)
University graduate	1.28 (0.24)	5.35 (0.0)	3.60	(2.24, 5.77)
Family history (Yes)	0.40 (0.23)	1.79 (0.08)	1.49	(0.96, 2.32)
Personal history (Yes)	0.84 (0.47)	1.80 (0.07)	2.32	(0.93, 5.77)
Attending in screening and educational program (Yes)	0.95 (0.32)	2.99 (0.003)	2.58	(1.39, 4.81)

^1^Class 2 was considered as reference category in LCR model.

^2^Reference: Less than 12 years formal education, less educated or illiterate.
